# Severe rise in tacrolimus levels following blood orange and grapefruit juice intake in a kidney transplant recipient: a case report

**DOI:** 10.1186/s13256-026-06514-w

**Published:** 2026-09-07

**Authors:** Victoria Östman, Anna-Karin Hamberg, Shinji Yamamoto, Tomas Lorant

**Affiliations:** 1 Department of Pharmacy, Uppsala University, Uppsala, Sweden; 2 Department of Surgical Sciences, Uppsala University, Uppsala, Sweden

**Keywords:** Tacrolimus, Food–drug interaction, CYP3A4 inhibition, Kidney transplantation, Case report

## Abstract

**Background:**

Tacrolimus is a cornerstone immunosuppressant in kidney transplantation with a narrow therapeutic index and high interindividual pharmacokinetic variability, largely determined by CYP3A4, CYP3A5, and P-glycoprotein activity. Grapefruit juice is a known intestinal CYP3A4 inhibitor capable of increasing tacrolimus exposure, whereas blood orange juice has not been documented to exert such effects.

**Case presentation:**

A 33-year-old male kidney transplant recipient of Somali origin had persistently low tacrolimus trough levels, averaging about 4.5 µg/L, despite increased dosing. On postoperative day 5 (POD 5), the patient consumed approximately one liter of blood orange juice followed later the same evening by a clinically supervised 250 mL dose of grapefruit juice intended to enhance tacrolimus exposure. The following morning, his tacrolimus concentration had risen sharply sixfold to 30 µg/L, far above the increase typically associated with grapefruit juice alone. Kidney biopsy at that time showed eosinophilic infiltration, consistent with a drug-related inflammatory reaction. Tacrolimus therapy was temporarily stopped, then gradually reintroduced. His condition stabilized, and he was discharged on POD 12.

**Conclusions:**

The pronounced increase in tacrolimus exposure following sequential ingestion of blood orange and grapefruit juice suggests a potential synergistic interaction affecting tacrolimus disposition via CYP3A4, CYP3A5 and/or P-glycoprotein pathways. While causality remains unconfirmed, this case emphasizes the value of thorough dietary history in transplant care and supports further research on blood orange juice as a possible modulator of tacrolimus pharmacokinetics.

## Background

Tacrolimus is a cornerstone immunosuppressive agent widely used in kidney transplant recipients. It has a narrow therapeutic index and high interindividual variability due to metabolism via CYP3A4, CYP3A5 and efflux by P-glycoprotein (P-gp) [1, 2]. Drug levels are significantly influenced by factors affecting these proteins, including genetic polymorphism, liver metabolic capacity, age, concomitant medications that inhibit or induce CYP3A4 or P-gp (e.g., ketoconazole, rifampicin, verapamil, St. Johns’ Wort), and dietary components, such as grapefruit juice [3–9]. Therapeutic drug monitoring is essential in transplant recipients to ensure efficacy while avoiding toxicity, particularly in the early post-transplant period [10].

In clinical practice, grapefruit juice is occasionally used as a pharmacokinetic enhancer in transplant patients with persistently low tacrolimus trough levels despite high dosing. This strategy aims to inhibit intestinal CYP3A4 and thereby increase drug bioavailability [8, 11, 12]. At the treating hospital, this approach is part of local clinical practice and is applied under close clinical supervision in selected patients. Based on clinical experience, supported by existing literature, the expected increase in tacrolimus exposure is approximately threefold [13]. However, a case report by Fukatsu et al. (2006) described a tenfold increase in tacrolimus trough concentration in a liver transplant recipient who consumed grapefruit juice [14]. Notably, in that case the elevation occurred 7 days after the final ingestion of grapefruit juice, with no immediate effect observed during the administration period.

While grapefruit juice is known to increase tacrolimus bioavailability via CYP3A4 inhibition, blood orange juice is not recognized for such effects. We present a case suggesting a clinically significant interaction between blood orange juice, grapefruit juice and tacrolimus.

## Case presentation

A 33-year-old male patient of Somali origin with end-stage renal disease due to hypertension and prior schistosomiasis underwent deceased-donor kidney transplantation. He received standard immunosuppressive induction therapy with basiliximab and was maintained on tacrolimus, mycophenolate mofetil, and corticosteroids. Despite dose escalation of immediate-release tacrolimus (from 3.5 mg twice daily (BID) to 10 mg BID), trough blood levels remained subtherapeutic (4.3–4.9 µg/L).

On postoperative day (POD) 4, liver biochemistry showed elevated transaminases, with AST 2.14 µkat/L and ALT 2.73 µkat/L, while alkaline phosphatase was 1.4 µkat/L, bilirubin 2.8 µmol/L, PT-INR 1.2, and aPTT 34 s, indicating mild hepatocellular injury but preserved synthetic liver function.

**Fig. 1:**
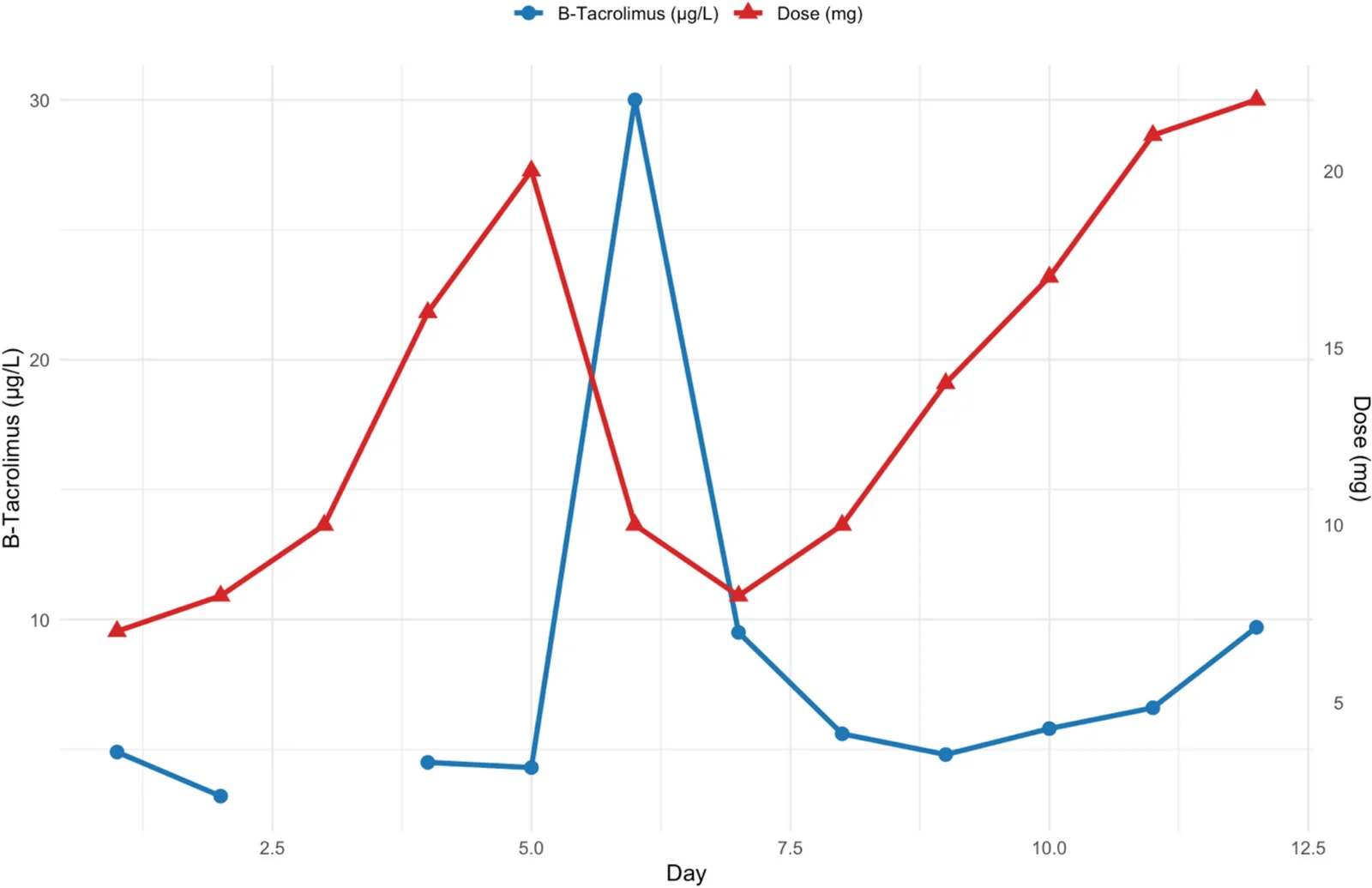
Tacrolimus trough concentrations (µg/L) and daily dose (mg) over postoperative days. A marked increase in tacrolimus concentration is observed on POD 6 following reported intake of blood orange juice and subsequent grapefruit juice administration

On POD 6, following a clinically supervised intake of grapefruit juice (~ 250 ml) on the evening of POD 5 to raise tacrolimus levels, a spike to 30 µg/L was observed. The temporal relationship between tacrolimus dosing and trough concentrations is illustrated in Fig. 1. A subsequent dietary history revealed the patient had consumed approximately one liter of blood orange juice earlier on the evening of POD 5. Serum creatinine was 253 µmol/L on the day the elevated tacrolimus concentration was detected. A transplant biopsy revealed eosinophilic infiltration suggestive of a drug-mediated inflammatory response without signs of rejection.

On POD 7, repeat liver biochemistry showed AST 1.74 µkat/L, ALT 3.35 µkat/L, alkaline phosphatase 1.8 µkat/L, and bilirubin 5 µmol/L. Serum creatinine was 239 µmol/L. No additional laboratory or imaging abnormalities were identified. No concomitant medications known to interact with tacrolimus had been introduced in the days preceding the rise in tacrolimus concentration.

Tacrolimus dosing was temporarily interrupted and gradually re-titrated, and the patient was discharged on POD 12 in stable condition with the tacrolimus dose 11 mg BID and a trough level of 9.7 µg/L without any fruit juice coadministration. At 1-year follow-up, the patient demonstrated stable graft function, with a serum creatinine of 106 µmol/L and urea of 6.4 mmol/L, and no proteinuria. Maintenance immunosuppression included extended-release tacrolimus 6 mg daily (trough concentration 4.5 µg/L), mycophenolate mofetil 750 mg twice daily, and prednisolone 5 mg once daily.

## Conclusions and discussion

This case highlights a potential novel interaction between blood orange juice, grapefruit juice and tacrolimus. Grapefruit juice contains furanocoumarins (notably bergamottin and 6′,7′-dihydroxybergamottin) that inhibit intestinal CYP3A4, leading to increased tacrolimus bioavailability [15, 16]. While some other citrus fruits, like Seville oranges, can have similar effects, blood orange juice has not been tested or shown to inhibit CYP3A4 in controlled studies. The absence of direct studies means that conclusions regarding blood orange juice’s effects on drug metabolism are speculative. The spike in tacrolimus level exceeded the expected effect of grapefruit juice alone. This implies that blood orange juice may potentially affect tacrolimus metabolism under specific conditions.

However, several factors may explain this case. A variability in juice composition, commercial or fresh-squeezed blood orange juice may vary widely in furanocoumarin content. The specific brand of commercial juice used in this case could not be verified retrospectively which limits assessment of its furanocoumarin content, but some commercial blood orange juice may inadvertently contain CYP3A4 inhibitors [17–19]. Genetic polymorphism in CYP3A4, CYP3A5, or P-gp that predispose to exaggerated pharmacokinetic responses to mild inhibition may also occur. CYP3A4, CYP3A5, and ABCB1 genotype data were not available in this case, which represents an important limitation. CYP3A5 expression is more common among individuals of African ancestry and is associated with lower tacrolimus exposure and higher dose requirements [20]. The absence of genotype data, therefore, limits ability to determine whether underlying genetic variability contributed to the observed pharmacokinetic response. Furthermore, the volume consumed (> 1L) may have unmasked mild inhibition that became clinically significant due to cumulative dosing. The blood orange juice could also have synergized with the later given grapefruit juice to produce an additive effect.

Other potential causes of the tacrolimus spike such as drug–drug interactions with other medications or impaired hepatic metabolism from liver dysfunction were considered. However, no medications interacting with tacrolimus was introduced in the days leading up to the spike in concentration and hepatic panel results around the time of the spike showed only mild transaminase elevations with preserved synthetic function [21]. These biochemical changes were mild and are unlikely, on their own, to account for the marked increase in tacrolimus exposure. The temporal relationship with the ingestion of large volumes of blood orange juice, in the context of prior grapefruit juice exposure, remains the most plausible explanation.

This case indicates that blood orange juice could potentially impact the disposition of drugs with narrow therapeutic indices that are substrates to CYP3A4, CYP3A5 or P-gp. It underscores the need for individualized dietary counseling and reveals a potential knowledge gap regarding the drug interaction potential of blood orange juice, particularly its possible synergistic inhibitory effects with grapefruit juice on intestinal enzymes.

## Data Availability

The data supporting the findings of this case report are not publicly available due to patient confidentiality but are available from the corresponding author upon reasonable request.

## Abbreviations

ALT: Alanine aminotransferase
ALP: Alkaline phosphatase
AST: Aspartate aminotransferase
aPTT: Activated partial thromboplastin time
BID: Twice daily (bis in die)
P-gp: P-glycoprotein
PT–INR: Prothrombin time–international normalized ratio
POD: Post-operative day
